# A Multidisciplinary Approach to the Management of Unusual Bilateral Gingival Recession Due to a Factitious Nail-Scratching Habit: A Case Report

**DOI:** 10.7759/cureus.53439

**Published:** 2024-02-02

**Authors:** Nasreen Ansari, Farrukh Faraz, Arundeep K Lamba, Shruti Tandon, Sachin Dhingra

**Affiliations:** 1 Periodontics, Maulana Azad Institute of Dental Sciences, New Delhi, IND

**Keywords:** amnion-chorion membrane, self-injurious behavior, nail-scratching habit, factitious injuries, gingival recession

## Abstract

Self-inflicted gingival injuries have been reported in children and adolescents as a result of multiple factors such as accidental trauma, fingernail biting, sucking on objects such as pencils, or a nail-scratching habit. Management of these lesions requires a multidisciplinary approach that includes psychotherapy, behavioral counseling, and definitive treatment of oral soft tissue lesions. This paper illustrates the diagnosis and treatment of a 16-year-old male patient with a bilateral gingival recession in mandibular canines due to habitual nail scratching. A multidisciplinary approach for treatment included behavior and psychological counseling, topical application of anesthetic & antimicrobial gels, and surgical management of gingival recession defects using a modified lateral pedicle flap with and without an indigenously prepared amnion-chorion membrane. Excellent soft tissue health was found at a two-year follow-up.

## Introduction

Self-injurious behavior (SIB), also known as factitious injury, self-mutilating injury, and injury resulting from masochistic habits, is the act of intentionally causing physical harm and suffering to oneself [1,2]. SIB is more frequently seen in people with emotional and psychological disturbances. It is also more common in children with intellectual disability, autism, schizophrenia, children belonging to stressful family situations, homeless people, drug addicts, and victims of sexual or physical abuse [3].

These self-inflicting injuries may result in a variety of clinical presentations and if not addressed promptly, may result in infection, pain, itchiness, ulceration, bleeding gums, gingival recession, and in some cases, advanced bone loss. Also, managing these cases poses a challenge when the underlying etiology is not identified and resolved. Treatment options for these types of injuries to oral tissues include addressing the underlying cause. Psychotherapy, behavior management through positive reinforcement, and maintaining a tranquil environment for such patients at home aid in the cessation of self-injurious behavior. Once the SIB is managed, the patient should be followed up at regular intervals and the residual periodontal defects can be managed at later stages.

This paper delineates a case of a factitious nail-scratching habit that leads to a bilateral gingival recession in mandibular canines. The patient was suffering from anxiety disorder due to academics and stressful family situations at home. Management involved psychological counseling and behavior modification along with the application of topical anesthetic and antimicrobial gels which was followed by surgical periodontal therapy for gingival recession coverage.

## Case presentation

A 16-year-old male patient reported to the Department of Periodontics, Maulana Azad Institute of Dental Sciences, New Delhi, with the chief complaint of itching, pain, and gum bleeding related to the lower front teeth. He noticed the start of redness and itching sensation restricted to lower front teeth, for six months. He also reported worsening of symptoms while eating and brushing which improved after rinsing with cold water. There was no history of systemic illness, medication, previous hospitalizations, and drug allergies. The patient denied having any deleterious oral habits. His parents reported that he was stressed and was not performing well in academics. Upon friendly conversation with the patient, he revealed that he has been stressed for the past one year because of ongoing family issues and was unable to perform well in school. He disclosed that he developed a habit of scratching his gums using fingernails for one year, following which he noticed continuous itching, pain, and gum bleeding, which made him want to scratch his gingiva even more.

**Figure 1:**
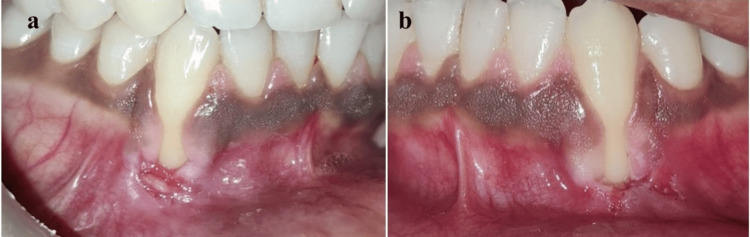
Ulcerated lesions at sites (a) 43 and (b) 33 due to self-inflicted trauma

On intraoral examination, bilateral Recession Type-1 (RT-1) defects (Cairo et al., 2011) were present in the mandibular right and left canines [4]. The involved gingiva was ulcerated, edematous, and covered with a white-yellowish membrane that was partially removed with the help of a moist gauze piece, leaving painful erythematous bleeding areas. The patient had good oral hygiene with minimal plaque deposits (Figure 1).

The patient was diagnosed with a self-injurious habit and was informed about the severity and possible sequelae of his condition. He was counseled regarding the damaging effects of his habit on the periodontium and was referred to a psychiatrist for the management of his apprehensive and self-injurious behavior. Parents were counseled to keep their home environment stress-free for the child.

**Figure 2:**
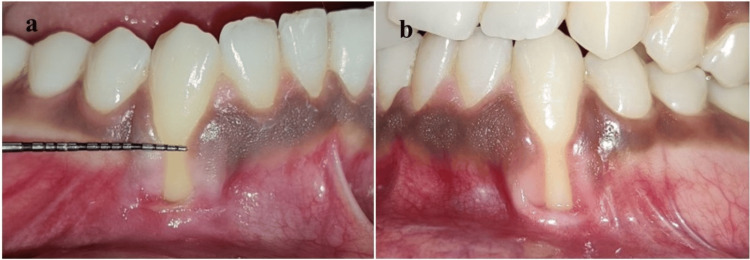
Complete healing after behavior counseling and phase-1 therapy at sites (a) 43 and (b) 33

At the initial visit, phase-I therapy was performed and the patient was prescribed a topical anesthetic and antimicrobial gel containing lignocaine, choline salicylate, and benzalkonium chloride (oral guard gel) to take a pea-size amount on a sterile applicator and apply twice daily on the affected area for 14 days. The patient was followed up after four weeks and reported with complete resolution of ulcerated lesions (Figure 2).

After four months of regular follow-up, periodontal maintenance therapy, and psychological counseling, root coverage surgery was planned using a modified lateral pedicle flap with and without an amnion-chorion membrane (ACM) at sites 33 and 43, respectively.

Baseline parameters recorded (in mm) before surgery using the UNC 15 probe (HUFRIEDY, USA) were probing depth (PD) (distance between the gingival margin and the bottom of the pocket), clinical attachment level (CAL) (distance between the cementoenamel junction (CEJ) and bottom of the pocket), keratinized tissue width (KTW) (distance between the most apical point of gingival margin and mucogingival junction), and recession height (RH) (measured from the CEJ to the most apical extension of the gingival margin at the mid-facial point of the teeth involved). The thickness of the Keratinized tissue (TKT) was measured 2 mm below the gingival margin of 33 and 43 using an endodontic spreader with a silicone disk stopper. The mucosa was pierced and the distance between the tip of the spreader and the inner border of the silicone stopper was measured to the nearest 0.1 mm with Vernier calipers (Table 1). The baseline plaque index score measured was 0.5 (Silness & Loe 1964) [5]. After obtaining written informed consent from parents, the surgical site was anesthetized using 2% lignocaine hydrochloride with 1:80,000 adrenaline.

**Table 1:** Baseline and follow-up data for tooth numbers 33 and 43 PD: Probing depth; CAL: clinical attachment level; RH: recession height; KTW: keratinized tissue width; TKT: thickness of keratinized tissue

Tooth number	Parameters recorded	Baseline	3 months	6 months	12 months
33	PD	1	1	1	1
	RH	7	0	0	0
	CAL	8	1	1	1
	KTW	1	6	7	7.5
	TKT	1.05	2.06	2.07	2.08
43	PD	1	1	1	1
	RH	6	0	0	0
	CAL	7	1	1	1
	KTW	1	6	6.5	7
	TKT	1.07	2.05	2.06	2.07

**Figure 3:**
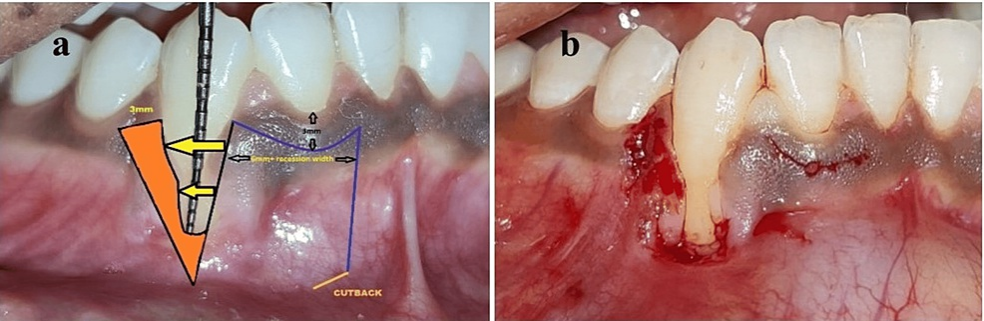
Surgical periodontal therapy to manage residual gingival recession defect: (a) Flap design and (b) incisions in place at site 43

The modified lateral pedicle flap was elevated using a 15c blade. A horizontal para-marginal incision was made at a distance of 8 mm mesial to the recession defect and 3 mm below the gingival margin of the lateral incisor, followed by the vertical incisions extending 5 mm beyond the mucogingival junction. The donor flap was elevated using a partial thickness dissection at the mesial and distal aspects and a full thickness dissection at the central portion of the flap which will cover the denuded root surface. The recipient bed was prepared distal to the recession defect using a 3 mm para marginal horizontal incision and an oblique vertical incision, followed by de-epithelization at the recipient site. To lessen the tension in the flap during lateral placement, a cut-back incision was made at the mesial end of the donor flap, toward the recipient site (Figure 3).

**Figure 4:**
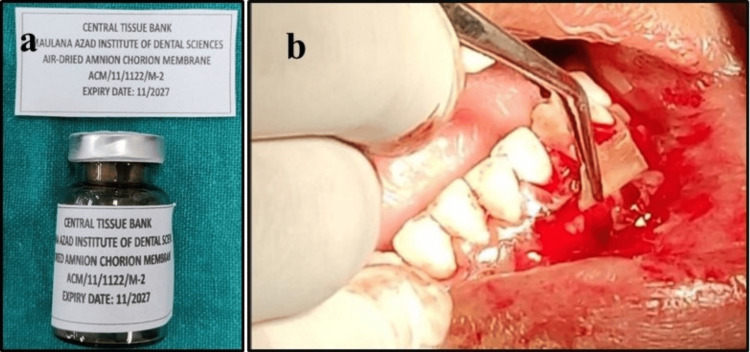
Surgical periodontal therapy to manage residual gingival recession defect: (a) Amnion chorion membrane and (b) amnion chorion membrane placed at site 33

The ACM (indigenously prepared at central tissue bank M.A.I.D.S using internationally acceptable standards) was placed at site 33 (Figure 4).

**Figure 5:**
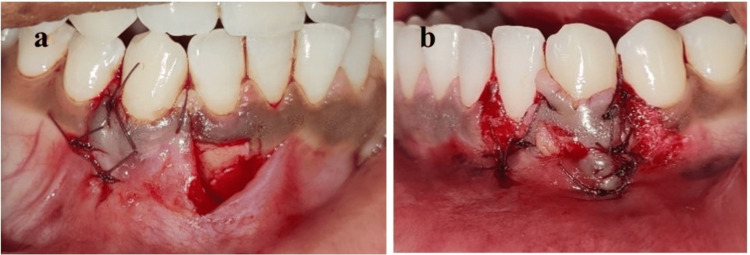
Lateral pedicle flap stabilized and secured with sutures at sites (a) 43 and (b) 33

The flaps were stabilized and sutured at their respective sites using interrupted 5-0 vicryl sutures (Figure 5).

**Figure 6:**
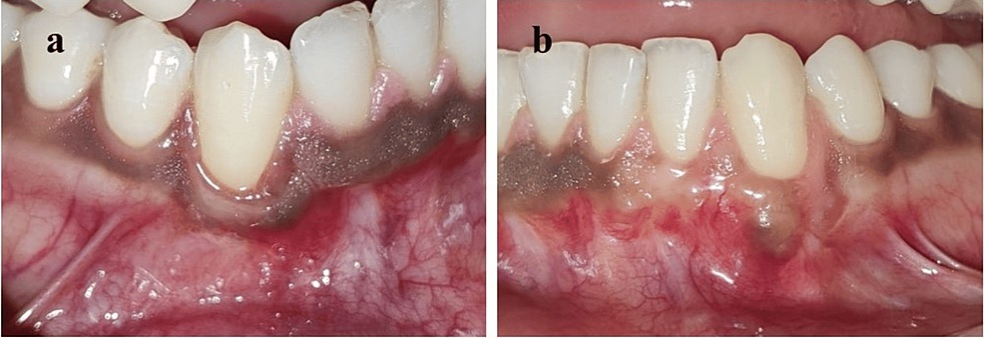
Postoperative view at 12-month follow-up (a) site 43 and (b) site 33

The patient was prescribed Ibuprofen 400 mg BD for three days and was advised to avoid brushing the surgical site for 14 days. Sutures were removed after 14 days and the patient was followed up for 3, 6, and 12 months. Complete root coverage was achieved at both sites with increased KTW (Figure 6).

A greater increase in the TKT was seen at site 33 where the ACM was placed as compared to site 43 where no graft membrane was applied (Table 1).

## Discussion

The self-inflicted trauma is more common in children than teenagers and adults [6]. This behavior can be attributed to severe anxiety, psychological and emotional disturbances, stress, and mental disorders. Among all the oral structures, the gingiva is most prone to injuries caused by SIB. Management of these lesions requires a multidisciplinary approach that includes correcting self-injurious behavior followed by managing residual lesions through surgical therapy if required.

This case study highlights the destructive nature of the factitious nail-scratching habit in a 16-year-old male patient and the stepwise approach for its successful treatment. In the present case, SIB in the patient leads to severe gingival recession along with ulceration. This behavior in the patient was attributed to his stressful family situation and poor academic performance. Dilsiz et al. (2009) reported a case of factitious nail-scratching habit in a 14-year-old female patient, causing gingival recession, which was managed using a free gingival graft (FGG) after psychological and behavioral counseling [7]. In the present case, a similar lesion was seen but to keep surgical therapy minimally invasive, the ACM was used instead of FGG. The modified lateral pedicle flap design was employed in the present case because it is minimally invasive and avoids the development of additional surgical sites, as seen in FGG. It also ensures no recession at the donor site and less flap tension due to the incorporation of cut-back incision [8]. John et al. (2013) reported a case of fingernail biting in a six-year-old female patient which led to gingival striping in maxillary molars, which was managed with habit-breaking appliance and topic anesthetic gel application [9]. Mostafa et al. (2017) reported a similar case of SIB in a 14-year-old female patient, where alloderm was used to treat bilateral gingival recession in the mandibular canine region [10]. In the present case, ACM was placed at site 33 which resulted in an additional gain in KTW and TKT as compared to site 43, where no membrane was placed (Table 1).

Placental allografts have been used in medicine for more than 100 years, with initial use in skin wound applications in the early 1900s [11]. In periodontal plastic surgery, placental allografts have recently evolved into a novel and versatile material. They possess unique inherent biological properties due to the presence of platelet-derived growth factor-AA (PDGF-AA), PDGF-BB, transforming growth factor α (TGF- α), TGF-β, fibroblast growth factor (FGF), epidermal growth factor (EGF) and IL-4, 6, 8, and 10, and tissue inhibitors of metalloproteinases (TIMP-1,2,3,4) which enhances cell proliferation, angiogenesis, metalloproteinase activity, and recruitment of progenitor cells [12]. High concentrations of these growth factors could accelerate wound healing and integration of the membrane with gingival tissue, thereby minimizing post-operative recession [13]. Minimal thickness and self-adherent properties of the ACM enable it to closely conform to contours around roots as well as above recession defects [14]. However, to formulate concrete evidence regarding the effectiveness of ACM in gingival recession coverage procedures, well-designed randomized controlled trials with larger sample sizes are required.

## Conclusions

Injurious oral habits in child patients and teenagers require prompt diagnosis and treatment as their persistence over a longer period complicates the treatment plan and can have a negative impact on physical and psychological health. The modified lateral pedicle flap design was chosen as it has several advantages such as preventing the gingival recession at the donor site, minimal invasiveness, and accelerated healing at the surgical site due to the pediculated donor flap. Also, the ACM used in this case provided additional benefits in terms of increased thickness of keratinized tissue at the site of its application and it may emerge as a promising graft material for the treatment of gingival recession defects.
